# Efficacy of endoscopic submucosal resection with a ligation device for small rectal neuroendocrine tumor: study protocol of a multicenter open-label randomized control trial (BANDIT trial)

**DOI:** 10.1186/s12876-024-03130-0

**Published:** 2024-02-08

**Authors:** Kazunori Takada, Kenichiro Imai, Takanori Yamada, Ken Ohata, Takashi Kanesaka, Yasuaki Nagami, Yasushi Yamasaki, Hideki Kobara, Yasuhiro Inokuchi, Akiko Chino, Shinjiro Yamaguchi, Hisatomo Ikehara, Takuji Kawamura, Yohei Yabuuchi, Yasuhiko Mizuguchi, Hiroaki Ikematsu, Chizu Yokoi, Santa Hattori, Kazuya Ohno, Yashiro Yoshizawa, Masakatsu Fukuzawa, Yosuke Tsuji, Jun Konishi, Takeshi Yamamura, Satoshi Osawa, Shiro Oka, Takuto Hikichi, Kazutomo Togashi, Kingo Hirasawa, Toshio Uraoka, Yoji Takeuchi, Hideyuki Chiba, Yoriaki Komeda, Hisashi Doyama, Mari S. Oba, Yutaka Saito

**Affiliations:** 1 Division of Endoscopy, Shizuoka Cancer Center, 1007 Shimonagakubo, NagaizumiShizuoka, 411-8777 Japan; 2 Department of Gastroenterology, Iwata City Hospital, Shizuoka, Japan; 3 Department of Gastroenterology, NTT Medical Center Tokyo, Tokyo, Japan; 4 Department of Gastrointestinal Oncology, Osaka International Cancer Institute, Osaka, Japan; 5 Department of Gastroenterology, Osaka Metropolitan University Graduate School of Medicine, Osaka, Japan; 6 Department of Gastroenterology, Okayama University Hospital, Okayama, Japan; 7 Department of Gastroenterology and Neurology, Faculty of Medicine, Kagawa University, Takamatsu, Japan; 8 Department of Gastroenterology, Kanagawa Cancer Center, Kanagawa, Japan; 9 Department of Gastroenterology, Cancer Institute Hospital of the Japanese Foundation for Cancer Research, Tokyo, Japan; 10 Division of Gastroenterology, Kansai Rosai Hospital, Hyogo, Japan; 11 Department of Gastroenterology, Kitasato University School of Medicine, Kanagawa, Japan; 12 Department of Gastroenterology, Kyoto Second Red Cross Hospital, Kyoto, Japan; 13 Department of Gastroenterology, Kobe City Medical Center General Hospital, Hyogo, Japan; 14 Endoscopy Division, National Cancer Center Hospital, Tokyo, Japan; 15 Department of Gastroenterology and Endoscopy, National Cancer Center Hospital East, Chiba, Japan; 16 Endoscopy Division, National Center for Global Health and Medicine, Tokyo, Japan; 17 Gastrointestinal Center, Sano Hospital, Hyogo, Japan; 18 Department of Gastroenterology, Shizuoka General Hospital, Shizuoka, Japan; 19 Department of Gastroenterology, Seirei Hamamatsu General Hospital, Shizuoka, Japan; 20 Department of Gastroenterology and Hepatology, Tokyo Medical University, Tokyo, Japan; 21 Next-Generation Endoscopic Computer Vision, Graduate School of Medicine, the University of Tokyo, Tokyo, Japan; 22 Department of Gastroenterology, Tochigi Cancer Center, Tochigi, Japan; 23 Department of Gastroenterology and Hepatology, Nagoya University Graduate School of Medicine, Aichi, Japan; 24 Department of Endoscopic and Photodynamic Medicine, Hamamatsu University School of Medicine, Shizuoka, Japan; 25 Department of Endoscopy, Hiroshima University Hospital, Hiroshima, Japan; 26 Department of Endoscopy, Fukushima Medical University Hospital, Fukushima, Japan; 27 Department of Coloproctology, Aizu Medical Center, Fukushima Medical University, Fukushima, Japan; 28 Division of Endoscopy, Yokohama City University Medical Center, Yokohama, Japan; 29 Department of Gastroenterology and Hepatology, Gunma University Graduate School of Medicine, Gunma, Japan; 30 Department of Gastroenterology, Omori Red Cross Hospital, Tokyo, Japan; 31 Department of Gastroenterology and Hepatology, Faculty of Medicine, Kindai University, Osaka, Japan; 32 Department of Gastroenterology, Ishikawa Prefectural Central Hospital, Ishikawa, Japan; 33 Department of Clinical Data Science, Clinical Research and Education Promotion Division, National Center of Neurology and Psychiatry, Tokyo, Japan

**Keywords:** Endoscopic submucosal dissection, Ligation, Neuroendocrine tumors, Randomized controlled trial, Resection margin

## Abstract

**Background:**

Endoscopic resection is widely accepted as a local treatment for rectal neuroendocrine tumors sized ≤ 10 mm. However, there is no consensus on the best method for the endoscopic resection of rectal neuroendocrine tumors. As a simplified endoscopic procedure, endoscopic submucosal resection with a ligation device (ESMR-L) indicates a histologically complete resection rate comparable to that of endoscopic submucosal dissection (ESD). We hypothesized that ESMR-L than ESD would be preferred for rectal neuroendocrine tumors. Hence, this trial aimed to verify whether ESMR-L is non-inferior to ESD in terms of histologically complete resection rate.

**Methods:**

This is a prospective, open-label, multicenter, non-inferiority, randomized controlled trial of two parallel groups, conducted at the Shizuoka Cancer Center and 31 other institutions in Japan. Patients with a lesion endoscopically diagnosed as a rectal neuroendocrine tumor ≤ 10 mm are eligible for inclusion. A total of 266 patients will be recruited and randomized to undergo either ESD or ESMR-L. The primary endpoint is the rate of en bloc resection with histologically tumor-free margins (R0 resection). Secondary endpoints include en bloc resection rate, procedure time, adverse events, hospitalization days, total devices and agents cost, adverse event rate between groups with and without resection site closure, outcomes between expert and non-expert endoscopists, and factors associated with R0 resection failure. The sample size is determined based on the assumption that the R0 resection rate will be 95.2% in the ESD group and 95.3% in the ESMR-L group, with a non-inferiority margin of 8%. With a one-sided significance level of 0.05 and a power of 80%, 226 participants are required. Assuming a dropout rate of 15%, 266 patients will be included in this study.

**Discussion:**

This is the first multicenter randomized controlled trial comparing ESD and ESMR-L for the R0 resection of rectal neuroendocrine tumors ≤ 10 mm. This will provide valuable information for standardizing endoscopic resection methods for rectal neuroendocrine tumors.

**Trial registration:**

Japan Registry of Clinical Trials, jRCTs042210124. Registered on Jan 6, 2022.

## Background

Neuroendocrine tumors (NETs) are rare, however, their incidence and prevalence are increasing globally [1]. The gastrointestinal tract is the leading primary site of NETs, particularly rectal NETs, which commonly occur among Asian/Pacific Islanders [1–3]. Considering the increasing prevalence of rectal NETs, effective management strategies are crucial in clinical practice. The management of rectal NETs depends on the tumor size and depth. NETs measuring ≥ 20 mm or showing invasion of the muscularis propria (MP) require radical surgery because of their high metastatic potential. The management of lesions sized 10–20 mm is debatable in the guidelines; however, this size category has been associated with a significant lymph node metastasis rate of 30–66% [3, 4]. In contrast, for rectal NETs measuring ≤ 10 mm, the rate of lymph node metastasis was 3%, and the 5-year cancer-specific survival rate was 100% in patients undergoing both local excision and radical surgery [5]. It is widely accepted that lesions sized ≤ 10 mm without MP invasion are suitable for local resection, with endoscopic resection being recommended in several guidelines due to its minimal invasion [6–8]. Reports indicate various methods for the endoscopic resection of rectal NETs, including polypectomy, conventional endoscopic mucosal resection (EMR), endoscopic submucosal dissection (ESD), modified EMR, such as cap-assisted EMR (EMR-C), and endoscopic submucosal resection with a ligation device (ESMR-L) [9–30]. The primary objective of the endoscopic resection of NETs is to achieve histologically complete resection (R0 resection) [8]. Previous meta-analyses have compared different techniques, such as modified EMR and ESD, with conventional EMR. However, it revealed varying results regarding the superiority of one method over the other in terms of R0 resection rates. One meta-analysis demonstrated the superiority of modified EMR to ESD (R0 rates:93.7% vs. 84.1%), whereas another reported no significant differences (94.4% vs. 91.8%) [31, 32]. However, these meta-analyses were based on retrospective studies and no multicenter randomized controlled trials (RCT) have directly compared these techniques. Therefore, the current evidence level remains low, as stated in the recent European Society of Gastrointestinal Endoscopy (ESGE) guideline [8]. Consequently, there is no consensus on the optimal method for endoscopic resection of rectal NETs. ESD is considered the most reliable resection technique for colorectal adenomas and superficial cancers, with a high R0 resection rate [33, 34]. However, ESD is associated with longer procedure times and requires expensive specialized equipment, whereas modified EMR is a simpler and less technically demanding approach [32]. Among the modified EMR, ESMR-L has shown a relatively higher R0 resection rate (89–100%) than EMR-C (83–92%) [30–32]. Given these considerations, if ESMR-L achieves R0 resection rates comparable with those of ESD, it may be preferred for rectal NETs.

The BANDIT trial aims to verify whether ESMR-L is inferior to ESD in terms of the R0 resection rate.

## Methods and design

### Study settings

**Fig. 1:**
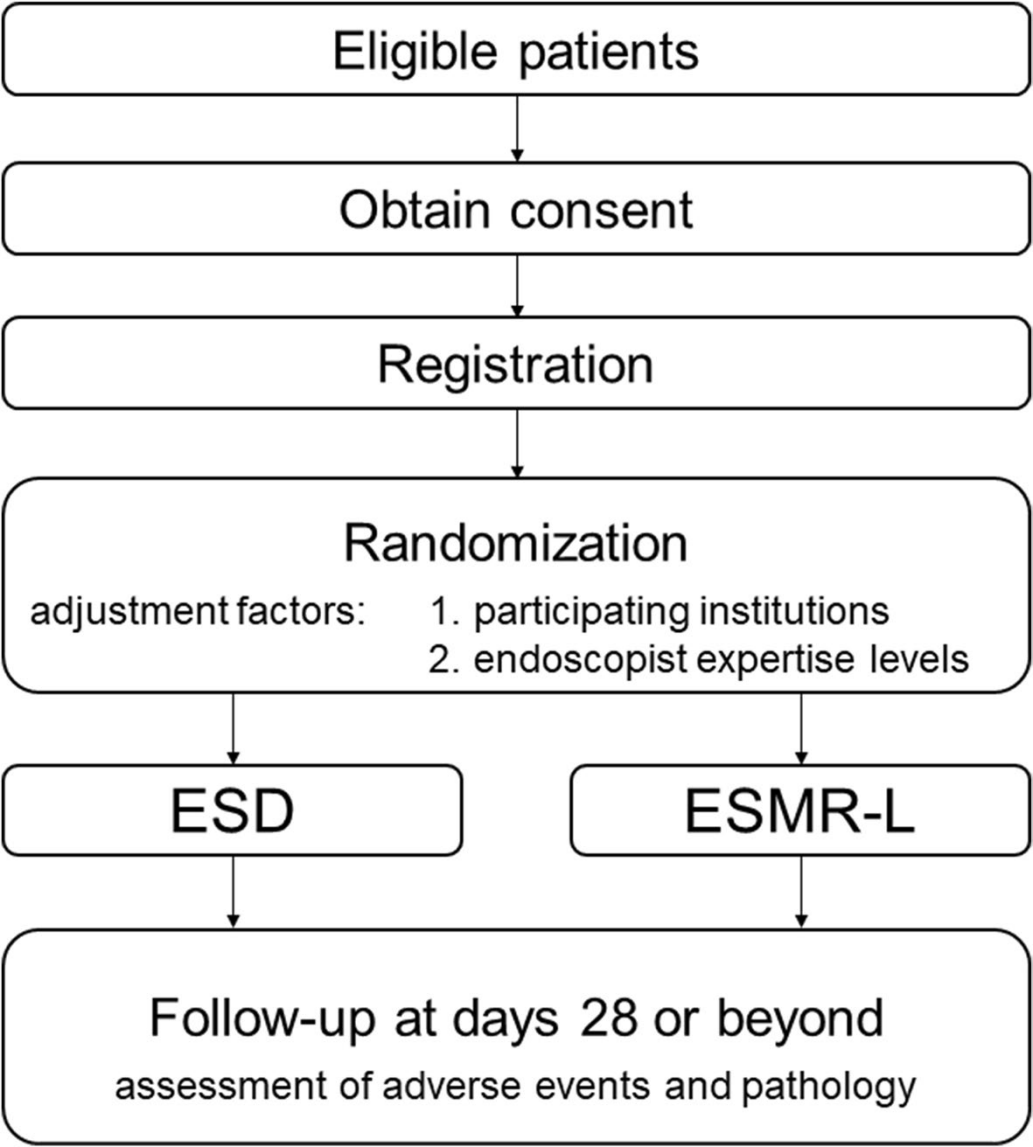
Flowchart of the study design. ESD, endoscopic submucosal dissection; ESMR-L, endoscopic submucosal resection with a ligation device

**Fig. 2:**
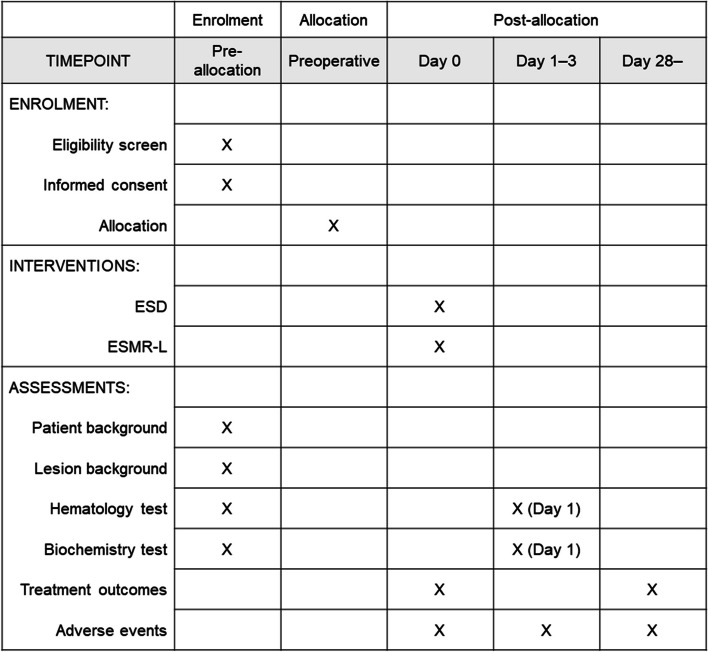
Schedule of enrollment, interventions, and assessments as per Standard Protocol Items: Recommendations for Interventional trials (SPIRIT). ESD, endoscopic submucosal dissection; ESMR-L, endoscopic submucosal resection with a ligation device

This is a prospective, open-label, multicenter, non-inferiority RCT of two parallel groups, conducted at the Shizuoka Cancer Center and 31 other institutions in Japan. A flowchart of the BANDIT trial is displayed in Fig. 1. The trial protocol was designed according to the Standard Protocol Items: Recommendations for Interventional Trials (SPIRIT) guidelines [35]. The SPIRIT flow diagram is shown in Fig. 2.

### Approvals

The study protocol was approved by the Certified Review Board (CRB) of Shizuoka Cancer Center (CRB4180010; Nov 24, 2021). The trial was registered in the Japan Registry of Clinical Trials (jRCTs042210124; Jan 6, 2022).

### Eligibility criteria

#### Inclusion criteria

Patients should meet all of the following criteria.

- 1) Patients with a lesion endoscopically diagnosed as rectal NET ≤ 10 mm.
- 2) Patients who are at least 20 years old at the time of obtaining consent.
- 3) Written informed consent should be provided based on the free will of patients after they have thoroughly understood the instructions provided regarding study participation.

#### Exclusion criteria

Patients are excluded if they met any of the following criteria.

- 1) Patients whose clinical course cannot be followed up to 28 days after treatment.
- 2) Patients with a recurrent lesion.
- 3) Patients with inflammatory bowel disease or colorectal polyposis.
- 4) Patients with coagulation dysfunctions.
- 5) Patients with severe infectious disease.
- 6) Patients on dialysis.
- 7) Patients who cannot discontinue antithrombotic agents based on the guidelines for gastroenterological endoscopy in patients undergoing antithrombotic treatment [36, 37].
- 8) Pregnant patients.
- 9) Other cases determined to be unfit for the study by the investigator.

### Informed consent procedure

Patients are screened for eligibility by endoscopists at each institution based on the above-mentioned criteria. The patients will receive detailed information about the trial from a member of the research team at their respective institutions. Ample time will be provided to patients for considering participation in the trial. Written informed consent will be obtained from each patient before enrollment.

### Randomization

Upon enrollment of eligible patients by the investigators, a randomization process will be conducted to assign patients to either the ESD or the ESMR-L group. This randomization will follow a 1:1 allocation ratio and will be performed using a web-based registration system available at Shizuoka Cancer Center, which is accessible 24 h a day. This study will use minimization method. Adjustment factors are participating institutions and endoscopist expertise levels (expert or non-expert).

### Blinding

Owing to the nature of the trial intervention, blinding endoscopists, and patients is not feasible. Endoscopists should be aware of the allocated group at the time of treatment, and patients may become aware of their assigned group during the course of treatment.

### Trial interventions

Each patient with a rectal NET is treated using the assigned endoscopic procedure. Only one target lesion is treated per patient. Hospitalization is recommended, however, it is not mandatory.

### Standard treatment group (ESD group)

ESD is a procedure involving a mucosal incision and submucosal dissection using ESD knives [38]. ESD knives are specified to the following; DualKnife J (KD-655, Olympus Medical Systems Corp, Tokyo, Japan), FlushKnife BT-S (DK2620J, Fujifilm, Tokyo, Japan), HookKnife J (KD-625, Olympus Medical Systems Corp), and Jet B-knife (Zeon Medical Inc, Tokyo, Japan). The endoscope is selected at the discretion of the operator. CO2 insufflation is recommended. This trial does not specify the type and mode of the electrosurgical unit, the use of a distal attachment, or the type of solution for submucosal injection. Traction devices are allowed to use. In cases of intraprocedural bleeding, hemostasis is achieved through coagulation with the tip of the ESD knife or hemostatic forceps. If intraprocedural perforation or muscle injury occurs, the resection site will be clipped.

### Trial treatment group (ESMR-L group)

ESMR-L will be performed using an endoscope equipped with a band ligator device (Pneumoactivate EVL device, SB-Kawasumi Laboratories, Inc, Tokyo, Japan) [9]. The lesion will be removed using submucosal injection. Subsequently, the lesion will be aspirated into the ligator device, followed by the deployment of the elastic band. Afterward, snare resection will be performed below the band using a blended current. The endoscope is selected at the discretion of the operator. CO2 insufflation is recommended. The type of solution used for the submucosal injection and the type of snare employed are not specified. In cases of bleeding immediately after resection, hemostasis is achieved by coagulation with the tip of the snare, hemostatic forceps, or clipping. If intraprocedural perforation or muscle injury occurs, the resection site will be clipped.

### Intervention success and failure

Treatment is considered successful when the operator determines that the specimen has been retrieved without any residual lesions. In cases where the operator deems it impossible to complete the resection using the assigned treatment and specified devices, the treatment protocol will be terminated. Additional treatment options may be allowed after termination.

### Restrictions after resection

In the absence of perforation or muscle injury, prophylactic clipping or covering methods, such as polyglycolic acid sheets and fibrin glue, are not recommended, however, they are permitted. If the operator applies prophylactic clipping or covering methods, they are required to apply the same method to both the ESD and ESMR-L groups. Prophylactic endoscopic coagulation is permitted only for visible vessels.

### Pathological assessment

Resected specimens are immediately fixed on a panel using pins in a manner that aligns with the tumor diameter. To evaluate the maximum cut surface and vertical margins, the specimens are fixed in 10% buffered formalin and sectioned at the deepest part of the tumor. Resected specimen size, tumor size, classification (NET G1, G2, G3, neuroendocrine carcinoma [NEC]), invasion depth, lymphatic invasion, vascular invasion, horizontal margins, and vertical margins are recorded. Final pathological diagnoses will be determined by pathologists at each participating institution utilizing the WHO Classification of Tumors, 5th Edition (2019) and the Japanese Classification of Colorectal Carcinoma [39].

Additional colectomy with lymphadenectomy may be considered for the following; tumor size ≥ 1 cm or grade higher than G2, positive vertical margins, muscularis propria invasion, or lymphovascular invasion [40].

### Outcome measures

#### The primary endpoint (for non-inferiority)

- 1. Rate of en bloc resection with histologically tumor-free margins (R0 resection rate)

R0 resection rate is defined as the absence of tumor involvement in both the horizontal and vertical margins, as confirmed histologically in the resected specimen. Evaluation of the horizontal and vertical margins should be performed on the plane where the NET or NEC is present. Margins without NET or NEC, irrespective of the distance, are considered to be tumor-free. Subsequently, R0 resection will be assessed by the operator based on a pathological report.

#### Secondary endpoints

- 2. En bloc resection rate

En bloc resection is defined as the complete removal of the lesion in a single piece without residual visible tumors.

- 3. Procedure time

Procedure time refers to the duration from submucosal injection to completion of the resection. It does not include the time required for prophylactic clipping, covering methods, or endoscopic coagulation of visible vessels after resection.

- 4. Adverse events (AEs)
  Adverse events will be monitored and recorded, including the following:
  - a. Delayed bleeding: bleeding that occurs within 28 days after colonoscopy withdrawal that requires hemostasis.
  - b. Intraoperative perforation: defect in the muscular layer observed during the procedure, or the presence of free air in the peritoneal cavity on imaging studies performed within 12 h after treatment.
  - c. Delayed perforation: bowel perforation within 28 days after the procedure without intraoperative perforation, confirmed by the presence of free air in the peritoneal cavity on imaging studies performed > 12 h after treatment.
  - d. Electrocoagulation syndrome: localized abdominal tenderness and fever (≥ 37.6℃), or inflammatory response without definite evidence of perforation.
- 5. Number of hospitalization days

Outpatient treatment will be considered as 0 days.

- 6. Total cost of the devices and agents

The costs associated with the ESD knife, band ligator device, injection needle, distal attachment, snare, traction device, clips, solution for submucosal injection, and antibiotics are calculated.

- 7. Rate of AEs among groups with and without closure of the resection site

Patients who undergo closure of the resection site using clips or detachable snares will be compared with those without closure.

- 8. Outcomes between expert and non-expert endoscopists

The outcomes of expert endoscopists, defined as those with experience in > 40 colorectal ESD procedures [38, 41]. will be compared with those of non-expert endoscopists.

- 9. Factors associated with failure of R0 resection

The potential factors contributing to the failure of achieving R0 resection, including treatment method, tumor location, tumor size, previous biopsy, and endoscopist expertise, will be analyzed.

### Serious adverse events

The following AEs are classified as serious: AEs resulting in death or posing a life-threatening risk, AEs requiring rehospitalization for treatment or extension of the hospitalization period, AEs leading to persistent or marked disability, and AEs with the potential to cause birth defects in the offspring. AEs will be assessed and graded according to the Common Terminology Criteria for Adverse Events Version 5.0. If a serious AE occurs, the investigators should report it immediately to the principal investigator. If the principal investigator determines a causal relationship between a serious AE and the protocol treatment, a serious AE report will be submitted to the CRB and the hospital director. In addition, information regarding the occurrence of serious AEs will be shared promptly with all the investigators.

### Sample size calculation

The sample size is calculated based on the primary outcome parameter, the R0 resection rate, to assess the non-inferiority of ESMR-L to ESD. These assumptions are based on previous studies and systematic reviews [18–32], with an expected R0 resection rate of 95.2% in the ESD group and 95.3% in the ESMR-L group. The non-inferiority margin is determined following the FDA Non-Inferiority Clinical Trials to Establish Effectiveness Guidance for Industry 2016 (https://www.fda.gov/regulatory-information/search-fda-guidance-documents/non-inferiority-clinical-trials). This FDA guidance illustrates the use of half the difference between the standard treatment and placebo as the non-inferiority margin. According to this guidance, the non-inferiority margin is set at 8% as half the superiority effect of the standard treatment (ESD, 95.2%) over the previous control arm (conventional EMR, the R0 resection rate 78% [29]). Based on these assumptions, 226 participants are required to assess the non-inferiority of the ESMR-L, with a one-sided significance level of 0.05 and a power of 80%. Considering a potential dropout rate of 15%, including patients without NET or NEC in the resected specimen and those who refused consent or were lost to follow-up, 266 patients will be included, with 133 patients in each arm. To achieve sufficient participant enrollment, 32 Japanese institutions will be included in this trial.

### Statistical analyses

The main analysis will be conducted using the full analysis set (FAS). The FAS is defined as the population of enrolled participants, excluding any of the following criteria: (1) any violation of the inclusion or exclusion criteria, (2) no treatment for the enrolled lesion, (3) no data after randomization, or (4) histologically no NET or NEC after treatment completion. Additionally, a complementary analysis will be conducted using the per-protocol set (PPS), which includes participants from the FAS who have completed the allocated treatment. Adverse events will be assessed in the population of enrolled participants who have undergone the allocated treatment and have been followed up for up to 28 days after the procedure.

The primary assessment parameters, R0 resection rate in each group, and differences between the groups are analyzed. The 90% CI for the difference will be calculated using the Miettinen–Nurminen method. To assess the non-inferiority of ESMR-L, the difference between the groups will be compared to the non-inferiority margin of 8% using a Farrington–Manning test with a one-sided alpha of 0.05. If non-inferiority is demonstrated, the superiority of ESMR-L will be examined using a one-sided alpha of 0.05.

The secondary assessment parameter, en bloc resection rate, will be analyzed using the same method as the primary endpoint. The procedure time, number of hospitalization days, and total cost of the devices and agents are compared using the Wilcoxon rank-sum test. The rates of adverse events between the groups with and without closure of the resection site will be compared using Fisher’s exact test.

A subgroup analysis by expert and non-expert endoscopists will be performed for the primary endpoint, rate of adverse events, and procedure time. Comparison between expert and non-expert endoscopists will be also performed. Factors associated with R0 resection failure will be analyzed using logistic regression.

### Data registration and management

The investigators will enter anonymized data into a web-based Electronic Data Capture (EDC) system hosted at the Shizuoka Cancer Center. The EDC system and associated databases are secured and protected by passwords. A dedicated data management team (data center) established by the director of the data management office at Shizuoka Cancer Center will be responsible for various data management tasks, including managing the data randomization system, EDC system management, EDC form design, data analysis, and verification. The aggregated data will be stored semi-permanently at the datacenter. When patients request data deletion upon withdrawal of consent, their data will be deleted.

### Monitoring

Monitoring will be performed to ensure the safety and accuracy of the trial in accordance with the protocol. Monitoring will be conducted centrally, based on the input data collected in the EDC system at the data center. In principle, monitoring is conducted annually. The monitoring personnel will verify that this trial is conducted in compliance with the Clinical Trials Act and the study protocol.

### Protocol amendments

In the case of the following protocol amendments, the principal investigator must notify the CRB of the amendments: (1) changes to the protocol or the informed consent document, (2) changes to the implementation plan, and (3) changes to the conflict of interest management criteria or plan. If there is a change in the implementation plan (jRCT registration content), other responsible physicians in each institution and the Minister of Health, Labor, and Welfare of Japan will be notified of protocol amendments.

### Compensation

This trial will join the clinical research insurance and compensate for the following payments according to the insurance contract: (1) the amount paid by the patient for the medical expenses incurred for the treatment of health damage, (2) a certain amount other than the medical expenses required for the treatment of health damage that requires hospitalization, and (3) compensation for death or residual disability (disability levels one through three). The principal investigator will assess the causal relationship between the treatment protocol of this trial and any health problems that occur.

### Dissemination policy

The progress and primary results of this trial will be disclosed on the jRCT platform. With the agreement of the principal investigator, the results of this trial will be disseminated in international peer-reviewed journals and presented at academic conferences. The confidentiality of the research participants will be strictly maintained when the results are disclosed. The datasets used and/or analyzed during the trial may be made available by the corresponding author and data center members upon reasonable request.

## Discussion

Several methods for the local excision of rectal NETs have been reported, including endoscopic resection by endoscopists and transanal surgery by surgeons. The goal of local NET excision is to achieve R0 resection. Considering local excision for rectal NETs ≤ 10 mm, several endoscopic resection methods have exhibited a high R0 resection rate; thus, transanal surgery may overkill such lesions. Among endoscopic resection methods, ESD and modified EMR have shown higher R0 resection rates than conventional EMR. Among the modified EMR, EMR-C and ESMR-L are commonly applied. EMR-C requires a dedicated transparent cap with an inner groove and a dedicated crescent-type snare. This was previously employed for resecting early esophageal cancer over the last decade, however, it is now unavailable in certain institutions [42]. ESMR-L relies on a band ligator device, commonly utilized for endoscopic variceal ligation, with any type of snare. The incidence of cirrhosis linked to esophageal variceal disease is 17 per 100,000 person-years [43]. which is approximately eight times greater than that of early esophageal cancer [44]. This implies broader accessibility of the band-ligator device compared with the dedicated devices for EMR-C. Here, we consider that the higher availability of ESMR-L increases the probability of its widespread application, superseding EMR-C. Although two meta-analyses have compared ESD and modified EMR, all the studies are retrospective, and no prospective study had been reported when we planned this RCT. Additionally, we believe that ESD may be overkill because it requires a longer procedure time, is more expensive, and is technically demanding compared with modified EMR. Therefore, if ESMR-L is inferior to ESD in terms of R0 resection, ESMR-L will become the standard treatment for rectal NETs ≤ 10 mm. One meta-analysis has shown the superiority of modified EMR over ESD [32]. Furthermore, we will examine the superiority of ESMR-L if non-inferiority is shown. To ensure the simplicity of ESMR-L, we assessed procedure time, adverse events, hospitalization days, and total cost. We expect that the outcomes of the expert and non-expert endoscopists will help assess the generalizability of these procedures.

Recently, a non-inferiority RCT comparing modified EMR-C (EMR-C without injection) and ESD for the treatment of rectal NETs ≤ 10 mm has been reported from China [45]. This single-center study included 38 modified EMR-C and 41 ESD cases. The histological complete resection rate was 97.4% and 92.7% in the modified EMR-C and ESD groups, respectively, confirming the non-inferiority of the modified EMR-C. Our study has several strengths compared with the previous study. First, this was a multicenter study conducted at 32 institutions in Japan, including expert and non-expert endoscopists. The universality and generality of the findings were confirmed by our study setting. Second, the number of included patients will be three times more in this study. The difference in sample size is mainly attributed to the differences in the non-inferiority margin. We set the non-inferiority margin to 8% based on the FDA non-inferiority clinical trials guidance, whereas a study from China set the non-inferiority margin to 15% [45]. A strict sample size calculation will lead to robust results. Third, the higher availability of ESMR-L increases the probability of its widespread application, superseding EMR-C. Hence, we believe that this BANDIT trial will provide valuable information for determining the standard treatment for rectal NETs ≤ 10 mm.

### Trial status

The first version of the protocol was approved on Nov 24, 2021. The 5th version was approved on Mar 6, 2023. The amendments include modifying cooperative researchers. Recruitment began on Jun 9, 2022, and is expected to end by Jul 2025.

## Data Availability

Access to the dataset will be limited to the data center members.

## Abbreviations

AE: Adverse event
CI: Confidence interval
CRB: Certified Review Board
EDC: Electronic Data Capture
EMR: Endoscopic mucosal resection
EMR-C: Cap-assisted endoscopic mucosal resection
ESGE: European Society of Gastrointestinal Endoscopy
ESD: Endoscopic submucosal dissection
ESMR-L: Endoscopic submucosal resection with a ligation device
FAS: Full analysis set
MP: Muscularis propria
NEC: Neuroendocrine carcinoma
NET: Neuroendocrine tumor
PPS: Per-protocol set
RCT: Randomized controlled trial
SPIRIT: Standard Protocol Items: Recommendations for Interventional Trials
